# Monocarboxylate Transporter 4 Triggered Cell Pyroptosis to Aggravate Intestinal Inflammation in Inflammatory Bowel Disease

**DOI:** 10.3389/fimmu.2021.644862

**Published:** 2021-05-19

**Authors:** Yaodong Wang, Xiaorong Zhou, Kejian Zou, Guanhua Chen, Ling Huang, Fangying Yang, Wenxu Pan, Hongwei Xu, Zhaohui Xu, Huan Chen, Jiayu Chen, Sitang Gong, Xuan Zhou, Wanfu Xu, Junhong Zhao

**Affiliations:** ^1^ Department of Gastroenterology, Kunshan Hospital of Traditional Chinese Medicine, Kunshan Affiliated Hospital of Nanjing University of Chinese Medicine, Kunshan, China; ^2^ Department of Respiratory and Critical Care, The Fifth Affiliated Hospital Sun Yat-Sen University, Zhuhai, China; ^3^ Department of General Surgery, Hainan General Hospital, Haikou, China; ^4^ Department of Gastroenterology, Guangzhou Women and Children’s Medical Center, Guangzhou Medical University, Guangzhou, China; ^5^ Department of Neonatal Intensive Care Unit, Guangzhou Women and Children’s Medical Center, Guangzhou Medical University, Guangzhou, China; ^6^ Guangzhou Institute of Pediatrics, Guangzhou Women and Children’s Medical Center, Guangzhou Medical University, Guangzhou, China; ^7^ Department of Hematology, Zhujiang Hospital, Southern Medical University, Guangzhou, China

**Keywords:** MCT4, caspase-1, cell pyroptosis, inflammatory bowel disease, NF-κB

## Abstract

NLRP3 inflammasome has emerged as a crucial regulator of inflammatory bowel disease (IBD) characterized by a chronic inflammatory disease of the gastrointestinal tract. The expression of MCT4 is significantly increased in intestinal mucosal tissue of IBD, which has been identified to regulate intestinal barrier function. However, the function of MCT4 in cell pyroptosis remained unknown. In this study, we have established a stable cell line with MCT4 overexpression in HT-29 and CaCO2 cells, respectively. Functional analysis revealed that ectopic expression of MCT4 in CaCO2 cells contributed to cell pyroptosis as evidenced by LDH assay, which is largely attributed to Caspase-1-mediated canonical pyroptosis, but not Caspase-4 and Caspase-5, leading to cleave pro-IL-1β and IL-18 into mature form and release mediated by cleaved GSDMD. Mechanically, MCT4 overexpression in HT-29 and CaCO2 cell triggered the phosphorylation of ERK1/2 and NF-*κ*B p65, while inhibition of MCT4 by MCT inhibitor *α*-Cyano-4-hydroxycinnamic acid (*α*-CHCA) in HT-29 and CaCO2 cells led to a significant downregulation of ERK1/2 and NF-*κ*B activity. What’s more, blockade of ERK1/2-NF-*κ*B pathway could reverse the promotion effect of MCT4 on IL-1β expression. Importantly, both MCT4 and Caspase-1, GSDMD were significantly increased in patients with IBD, and a positive clinical correlation between MCT4 and Caspase-1 expression was observed (p < 0.001). Taken together, these findings suggested that MCT4 promoted Caspase-1-mediated canonical cell pyroptosis to aggravate intestinal inflammation in intestinal epithelial cells (IECs) through the ERK1/2-NF-*κ*B pathway.

## Introduction

Inflammatory bowel disease (IBD), including ulcerative colitis (UC) and Crohn’s disease (CD), is characterized by aberrant inflammation in the gastrointestinal tract accompanied with programmed necrosis and cell death (1, 2). A growing body of evidence indicated that dysregulated inflammasome activity intimately contributed to chronic intestinal inflammation, such as that observed in IBD (3–6), which was a critical platform that integrated environmental stimuli, including the exposome, the genome, the microbiome, and the immune system, to the main pathogenic event of IBD (7–10). Recently, cell pyroptosis-mediated epithelial-derived IL-1 and its family members played an essential role in the pathogenesis of IBD (11, 12), which was widely recognized and accepted (13).

Cell pryoptosis has been demonstrated to be associated with immune dysregulation and excessive inflammation response, resulting in serials of inflammatory diseases, including IBD (14–16). Generally, inflammasomes, comprised of an NLR(s), the adaptor protein ASC [apoptosis-associated speck-like protein containing a C-terminal caspase recruitment domain (CARD)] and Caspase-1, in the cytosol that recognizes PAMPs and DAMPs, which leads to the proteolytic activation of caspase-1. Active caspase-1 processed the maturation of pro-interleukin (IL)-1β and pro-IL-18 into mature and bioactive cytokines (17). Moreover, activated caspase-1 cleaves gasdermin D (GSDMD) into gasdermin-D N-terminal domain (GSDMD-N) and gasdermin-D C-terminal domain (GSDMD-C), while activated GSDMD-N formed pores in the cell membrane, and mature IL-1β and IL-18 are sequentially released outside of the cell (18). In addition to Caspase-1-induced canonical pyroptosis, both caspase-4 and caspase-5-mediated non-canonical pyroptosis released mature pro-inflammatory factors (19). These extracellular inflammatory cytokines may cause serious inflammation. Several studies have shown that IL-1β and IL-18 are the main inflammatory cytokines responsible for UC induction. However, the regulation of cell mechanism has remained to be elucidated.

It is well known that metabolic reprogramming played a critical role in intestinal barrier function. For instance, ketogenic mitochondrial enzyme hydroxymethylglutaryl CoA synthase 2 (HMGCS2)/ketone body *β*-hydroxybutyrate (*β*HB)-mediated ketogenesis contributed to intestinal cell differentiation by enhancement of caudal-related homeobox transcription factor 2 (CDX2) expression (20). AMP-activated protein kinase (AMPK), a master regulator of energy metabolism, also improved gut epithelial differentiation and barrier function through histone modifications in the CDX2 promoter (21). In addition, we have previously demonstrated the monocarboxylate transporter 4 (MCT4) is evaluated in intestinal mucosal tissue of patients with IBD (22); further study demonstrated that the overexpression of MCT4 in intestinal epithelial cells (IECs) could significantly reduce cAMP-response element binding protein (CREB)-mediated ZO-1 expression, while it promotes NF-κB binding to IL-6 promoter, leading to induce IL-6 secretion (23). In this study, we revealed a novel role of MCT4 in cell pyroptosis by triggering NLRP3 inflammasome form, resulting in IL-1β secretion to aggravate intestinal inflammation.

## Materials and Methods

### Chemical and Reagents

*α*-Cyano-4-hydroxycinnamic acid (*α*-CHCA) (C2020) was obtained from Sigma (St. Louis, MO, USA). BAY 11-7085 (HY-10257) was purchased from MedChemExpress, and U0126 (9903) was from Cell Signaling Technology (Danvers, MA, USA). Medium and fetal bovine serum (FBS) were purchased from Thermo Scientific (Kalamazoo, MI, USA). LDH (ab102526) was purchased from Abcam. Trizol was from Invitrogen (Invitrogen, Thermo Fisher Scientific). Antibodies were obtained from Abclonal. All-in-one™ first-strand cDNA synthesis kit and All-in-one™ qPCR mix were from Genecopoeia™ (Rockville, MD, USA). Other chemical reagents were from Sigma (St. Louis, MO, USA).

### Cell Culture

As described in Wang et al.’s study (20), CaCO2 and HT29 cell have served as *in vitro* model to delineate potential function of MCT4 in intestinal enterocytes (characterized by expression of the brush-border enzymes IAP and SI, villin, and Keratin 20 (KRT20) in CaCO2 and HT-29 cells) (24–26), goblet cells (characterized by the increased MUC2 expression in HT29) (27, 28), and Paneth cells as shown by the induction of LYZ in HT29 cells (29). HT-29 and CaCO2 cells were purchased from China Center for Type Culture Collection (Beijing, China) and maintained in DMEM (Thermo Scientific) with 10% fetal bovine serum (FBS) (Gibco, USA) in a humidified 5% CO_2_ incubator.

### Construction of Stable Cell Lines

As described in our pervious study (23), lentivirus of MCT4 overexpression was purchased from GenePharma (Shanghai, China) and used to infect HT-29 and CaCO2 cells to establish stable cell lines. Puromycin was from Sigma and used to select for stable cell line.

### LDH Assay

Cells were harvested and reseeded into 96-well plate. After 24 h, the LDH level was assessed by LDH assay. The optical absorbance at 490 nm was detected. The absorbance value of RPMI 1640 medium served as benchmark, and the absorbance value of completely lysed cells was regarded as the maximal LDH release. The practical value detected by LDH assay was corrected to indicate cytotoxicity.

### ELISA Assay

As described in our pervious study (23), the level of IL-1β was measured IL-18 in the serum and in the supernatants from HT-29 and CaCO2 cells with or without MCT4 overexpression which were collected and quantitated by sandwich ELISA according to the manufacturer’s instructions (Elabscience Biotechnology).

### RNA Extraction and Real-Time PCR Analysis

As described in our previously study (30), total RNA was extracted in indicated cells using trizol reagent (Invitrogen) according to the manufacturer’s protocol, and All-in-One First-Strand cDNA Synthesis Kit was used to convert mRNA into cDNA. The relative gene expression was performed for detection using an All-in-One qPCR Mix on an Applied Biosystems StepOnePlus system. Sequences of the primers used in this study are as follows: IL-1β:Forward: 5′-ATGATGGCTTATTACAGTGGCAA-3′, Reverse: 5′-GTCGGAGATTCGTAGCTGGA-3′; IL-18: Forward: 5′-TCTTCATTGACCAAGGAAATCGG-3′, Reverse: 5′-TCCGGGGTGCATTATCTCTAC-3′; GSDMD: Forward: 5′-GTGTGTCAACCTGTCTATCAAGG-3′, Reverse: 5′-CATGGCATCGTAGAAGTGGAAG-3′; NLRP3: Forward: 5′-GATCTTCGCTGCGATCAACAG-3′, Reverse: 5′-CGTGCATTATCTGAACCCCAC-3′; ASC: Forward: 5′-TGGATGCTCTGTACGGGAAG-3′, Reverse: 5′-CCAGGCTGGTGTGAAACTGAA-3′; Caspase-1: Forward: 5′-CCTTAATATGCAAGACTCTCAAGGA-3′’, Reverse: 5′-TAAGCTGGGTTGTCCTGCACT-3′; UBC: Forward: 5′-ATTTGGGTCGCGGTTCTTG-3′ and reverse, 5′-TGCCTTGACATTCTCGATGGT-3′.

### Western Blotting

Protein extraction and western blot analysis were performed as described (23). Proteins were separated by 10% SDS-PAGE and transferred to PVDF membrane (Millipore, USA) after blocking in PBS with 5% bovine serum albumin (BSA) for 1 h at room temperature. Primary antibodies were used to probe blots against indicated protein, which were incubated overnight at 4°C. HRP-conjugated secondary antibodies, including anti-rabbit and anti-mouse antibodies, were incubated for another 1 h at room temperature subsequently. The bands were visualized using a chemiluminescent imaging system.

### Immunofluorescence

Immunofluorescence was performed as in our pervious study (23); intestinal tissues were drawn from patients diagnosed with IBD and fixed in 10% paraformaldehyde. Briefly, the slides were dewaxed and rehydrated in distilled water, sections were immersed in citrate buffer (C_6_H_5_Na_3_O_7_·2H_2_O) and then microwaved for 20 min for antigen retrieval. Endogenous peroxidase activity was blocked with 0.5% (v/v) H_2_O_2_. Following blocking with 5% goat serum for 30 min, sections were incubated overnight (4°C) with different primary antibodies. After washing for three times, the section was incubated with appropriate fluorescent secondary antibodies and mounted using DAPI and imaged by fluorescent microscopy.

### Fluorescence Intensity Analysis

As described in the study (31), Image J software was used to calculate mean fluorescence intensity. To perform the image segmentation, the histogram of each image was first extended to saturate the gray scale. Then, the images were filtered using a median filter (r = 0.5). Afterwards 12-bit images were converted to an 8-bit gray scale. An algorithm then attributed pixels to the appropriate class of luminosity (255 steps of luminosity) which was arbitrarily divided into: class 1: 1 to 24; class 2: 25 to 50; class 3: 51 to 75; class 4: (the brightest): 76 to 100.

### Statistical Analysis

All statistical analysis and graphing were finished using the GraphPad Prism 8 software. The t test was used to determine the significance of differences in the qPCR assay, ELISA assays and LDH assay. One-way ANOVA was performed to determine the significance among groups. A p value of less than 0.05 was considered statistically significant.

## Results

### Overexpression of MCT4 in IECs Triggered GSDMD Activation, IL-1β and IL-18 Release

**Figure 1:**
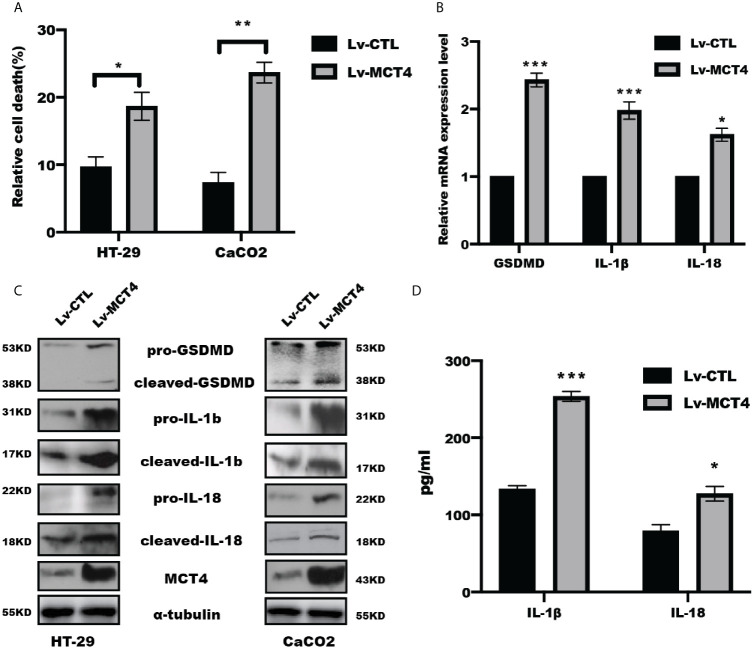
MCT4 triggered cell pyroptosis. **(A)** LDH assay was performed to determine relative cell death in indicated group in HT-29 and CaCO2 cell; data represented the mean ± s.d. of three independent experiments; t tests were used to analyze statistical significance; *p < 0.05, **p < 0.01. **(B)** Real-time PCR was performed to examine the mRNA level of GSDMD, IL-1β and IL-18 expression in CaCO2 cells. Data represented the mean ± s.d. of three independent experiments; t tests were used to analyze statistical significance; ***p < 0.001, *p < 0.05. **(C)** Western blotting was performed to test pyroptosis-related proteins, including GSDMD, IL-1β, and IL-18, in HT-29 and CaCO2 cells with or without MCT4 overexpression. **(D)** The contents of IL-1β and IL-18 in supernatant of HT-29 and CaCO2 cells with or without MCT4 overexpression were detected by ELISA assay; data represented the mean ± s.d. of three independent experiments; t tests were used to analyze statistical significance; ***p < 0.001, *p < 0.05.

We previously showed that overexpression of MCT4 in IECs destroyed intestinal barrier function and induced intestinal inflammation to aggravate IBD (23). To further address whether MCT4 is involved in the cell pyroptosis, we performed WB, real-time PCR, and ELISA assay to analyze the effect of MCT4 on the executor and production of pryoptosis, including GSDMD, IL-1β, and IL-18. The results showed that overexpression of MCT4 in IECs significantly increased relative cell death as evidenced by LDH assay (**Figure 1A**), and real-time PCR assay showed that GSDMD, IL-1β, and IL-18 expression at mRNA level (**Figure 1B**). In line with this, the results from WB further confirmed that ectopic expression of MCT4 drastically increased cleaved GSDMD, mature IL-1β and IL-18 expression in HT-29 and CaCO2 cells (**Figure 1C**). In addition, the ELISA result also demonstrated that ectopic expression of MCT4 in HT-29 and CaCO2 cells led to a significantly enhanced IL-1β and IL-18 release (**Figure 1D**). These results suggested MCT4 could promote cell pyroptosis in HT-29 and CaCO2 cells.

### MCT4 Contributed to Cell Pyroptosis Through NLRP3 Inflammasomes Form

**Figure 2:**
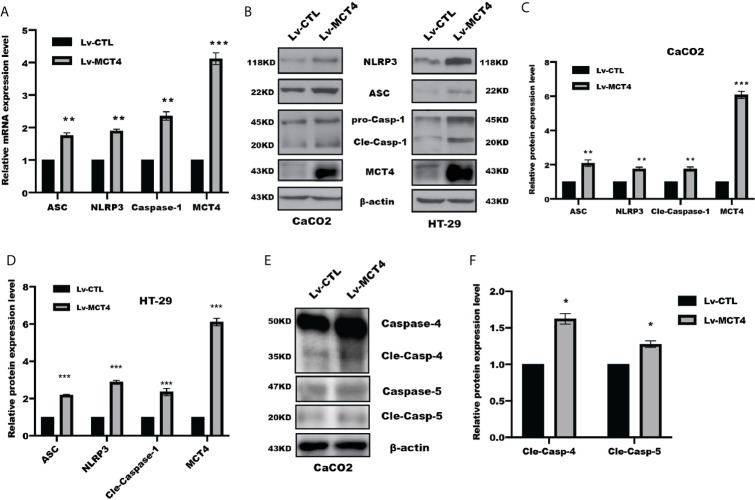
MCT4 promoted NLRP3 inflammasome activation. **(A)** Real-time PCR was performed to analyze the indicated gene expression in CaCO2 cells with or without MCT4 overexpression; data represented the mean ± s.d. of three independent experiments; t tests were used to analyze statistical significance; ***p < 0.001, **p < 0.01, n = 3. **(B)** Western blotting was used to detect NRLP3, ASC, Caspase-1, and MCT4 expression in the indicated group of HT-29 and CaCO2 cells. MCT4 was used to confirm the overexpression efficiency; *β*-actin served as internal control. The expression of the indicated proteins was assessed and quantified in CaCO2 **(C)** and HT-29 cells **(D)**. Data represented the mean ± s.d. of three independent experiments; t tests were used to analyze statistical significance; ***p < 0.001, **p < 0.01, n = 3. **(E)** Immunoblotting of Caspase-4 and Caspase-5 was performed in CaCO2 cells with or without MCT4 overexpression. **(F)** The expression of indicated proteins was assessed and quantified; data represented the mean ± s.d. of three independent experiments; t tests were used to analyze statistical significance, *p < 0.05.

The above results suggested MCT4 has a critical role in cell pyroptosis, which focused us to test the possible change of NLRP3 inflammasomes in response to MCT4 stimulation. As expected, real-time PCR assay revealed that overexpression of MCT4 in HT-29 and CaCO2 cells resulted in a significantly increased NLRP3, ASC, and caspase-1 mRNA (**Figure 2A**); in line with this, WB and intensity quantified results also showed that overexpression of MCT4 enhanced NLRP3, ASC, and caspase-1 expression at the protein level (**Figures 2B–D**), which in turn processed and released the inflammatory cytokines IL-1β and IL-18 by directly promoting pyroptosis through cleavage of the pore-forming protein GSDMD. What’s more, the slight change of cleaved caspase-4 and caspase-5 expression (**Figures 2E, F**) was observed. Taken together, our results showed MCT4 triggered cell pyroptosis in a caspase-1-dependent pathway.

### MCT4 Promoted NLRP3 Inflammasomes Through ERK1/2-NF-*κ*B Pathway in IECs

**Figure 3:**
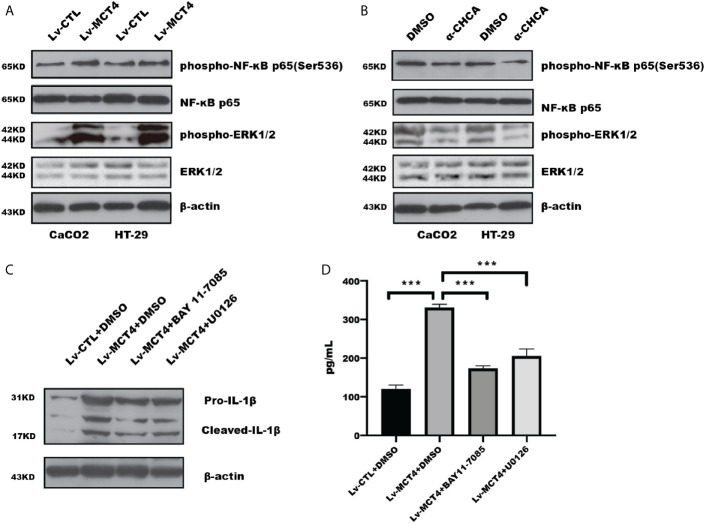
MCT4 regulated ERK1/2-NF-*κ*B pathway. **(A)** Western blotting was employed to test phosphorylation of NF-*κ*B and ERK1/2 activity in HT-29 and CaCO2 cells. **(B)** The total protein was collected in HT-29 and CaCO2 cells with or without *α*-CHCA stimulation for 1 h, and the indicated proteins was tested by immunoblotting. The ERK1/2 inhibitor U0126 and NF-*κ*B inhibitor BAY11-7085 were used to treat CaCO2 cells with MCT4 overexpression for 48 h. Western blotting **(C)** and ELISA **(D)** were used to analyze IL-1β expression and secretion, respectively. Data represented the mean ± s.d. of three independent experiments; one-way ANOVA was performed to analyze statistical difference. ***p < 0.001.

Our previous study have demonstrated that MCT4 expression was increased in intestinal mucosal tissue of IBD (22), and overexpression of MCT4 in IECs destroyed CREB-mediated ZO-1 expression and promoted NF-*κ*B-induced IL-6 expression (23). While *α*-Cyano-4-hydroxycinnamic acid (*α*-CHCA), a classical MCT inhibitor, has been reported to inhibit proliferation and induce p38-mediated apoptosis in murine pancreatic adenocarcinoma cell line 6606PDA and 7265PDA (32), which focused us to explore the potential mechanism through which MCT4 regulated cell pyroptosis. Our results showed that MCT4 overexpression in CaCO2 cell significantly increased phosphorylation of ERK1/2(p44/42) and NF-*κ*B p65(Ser536) (**Figure 3A**), while *α*-CHCA treatment caused an inhibition of ERK1/2 and NF-*κ*B activity (**Figure 3B**).

To further determine that phosphorylation of ERK1/2 and NF-*κ*B was involved in MCT4-mediated pyroptosis, we analyzed whether inhibition of ERK1/2 and NF-*κ*B activation could rescue the effect of MCT4 on pyroptosis. As shown in **Figures 3C, D**, NF-*κ*B inhibitor BAY 11-7085 and U0126, an inhibitor of ERK1/2, could reverse the promotion effect of MCT4 on IL-1β expression, which is attributed to the disruption of NLRP3 inflammasomes’ form.

### Clinical Relationship Between MCT4 Expression and Caspase-1 in Tissue Specimens

The above results suggested the critical role of MCT4 in cell pyroptosis, so we further focused on exploring the clinical relationship between MCT4 and pyroptosis-related proteins. As shown in **Table 1**, we have collected 45 subjects, including 30 patients with IBD and 15 healthy controls with aged between 1 and 16 years in Guangzhou Women and Children’s Medical Center. The study was divided into 15 healthy control subjects, consisting of 11 boys and four girls, and 30 patients with IBD, including 13 boys and 17 girls, respectively. Detailed clinical characteristics of the subjects, which are not public, could be available upon reasonable request.

**Table 1:** The characteristics of subjects enrolled in the study.

Variables	Number of subjects (%)	
Total	Control	IBD
	15(33.3%)	30(66.7%)
Age		
<10	7(15.6%)	16(35.6%)
>=10	8(17.8%)	14(31.0%)
Gender		
boy	11(24.4%)	13(28.9%)
girl	4(8.9%)	17(37.8%)
Type of IBD		
Crohn’s disease		22(73.3%)
Ulcerative colitis		8(26.7%)
Stage		
Active stage	0	20
Inactive stage	15	0

**Figure 4:**
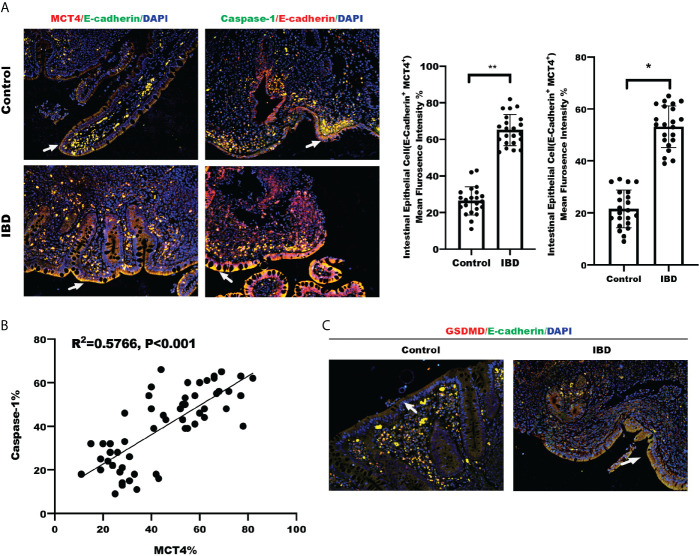
Clinical relationship between MCT4 and Caspase-1. **(A)** Immunofluorescence of MCT4 and Caspase-1 expression in intestinal epithelial cells labeled E-cadherin in Control group (n = 24) and IBD group (n = 24), respectively; the relative immunofluorescence intensity of MCT4^+^E-Cadherin^+^ and Caspase-1^+^E-Cadherin^+^ in intestinal epithelial cells was quantified by Image J and analyzed by t test. Data represented the mean ± s.d., **p < 0.01, *p < 0.05. **(B)** Clinical correlation between MCT4 and Caspase-1 expression was generated and analyzed by simple linear regression. **(C)** IF of GSDMD expression in intestinal epithelial cells labeled E-cadherin in Control group (n = 24) and IBD group (n = 24).

We detected MCT4 and Caspase-1 expression in a set slide tissue by IF to analyze their possible relationship with in IBD. The results showed that both MCT4 and Caspase-1 expression levels were increased in the intestinal epithelial cells labeled with E-cadherin in patients with IBD (**Figure 4A**). Further analysis of fluorescence intensity showed that MCT4 is positively correlated with Caspase-1 (**Figure 4B**). Also, we have detected GSDMD expression, a substrate of Caspase-1, in healthy control and IBD. The results showed that GSDMD is upregulated in IBD compared with that in healthy control (**Figure 4C**). Taken together, these findings suggested that both MCT4 and cell pyroptosis related protein, including caspase-1 and GSDMD, were increased in IBD, and MCT4 contributed to cell pyroptosis through caspase-1-mediated GSDMD activation.

## Discussion

Previous studies have reported that MCT4 expression was increased in intestinal mucosa in patients with IBD and contributed to the development of IBD by inhibition of ZO-1 and induction of IL-6 (22, 23). Herein, we further demonstrated that ectopic expression of MCT4 in IECs triggered cell pyroptosis in IECs, aggravating intestinal inflammation through ERK1/2-NF-*κ*B axis-mediated NLRP3 inflammasome activation, leading to caspase-1 activation, mature IL-1β and IL-18 release. Inhibition of ERK1/2-NF-*κ*B activity could overcome the effect of MCT4 on cell pryoptosis. What’s more, a significant upregulated expression of MCT4, Caspase-1, and GSDMD were observed in IBD tissue samples. These findings suggested that MCT4 could aggravate intestinal inflammation by cell pyroptosis.

Cell pyroptosis is involved in a variety of inflammatory disease and has gained more and more attention. It has been reported that the cytosolic Nod-like receptors are key determinants of health and disease. Nod-like receptors trigger inflammation by engaging NF-*κ*B and MAPK pathways or by scaffolding a caspase-1 inflammasome, required for the production of IL-1β and IL-18, which is crucial to maintain intestinal homeostasis (6). In this work, we have addressed that MCT4 contributed to cell pyroptosis by triggering Caspase-1 activation, leading to cleave GSMDM, mature IL-1β and IL-18 secretion to aggravate intestinal inflammation, which is line with the previous study indicated the pro-IBD function of MCT4 (23). Apart from caspase-1-mediated canonical pyroptosis, caspase-4-mediated non-canonical pyroptosis, not caspase-5, was also involved in MCT4-mediated pyroptosis in CaCO2 cells. Furthermore, the results of IHC analysis showed that both MCT4 and Caspase-1 expression were significantly increased in a set of intestinal mucosal tissue with IBD patients. However, further work is required to address the metabolic switch in IECs with MCT4 overexpression, and the effect of MCT4-mediated lactate acid in cell pyroptosis remained to be identified in future work.

The current studies have shown that mTOR/STAT3 axis (33) and IKK-NF-*κ*B pathway (34, 35), including IKK epsilon (36), played a critical role in regulating cell pyroptosis. In addition, a missense mutation of NLRP3 could lead to NLRP3 inflammasome activation causing cell pyroptosis (37–41), such as R779C increased NLRP3 inflammasome activation and pyroptosis in macrophages, which was mediated by enhanced deubiquitination of NLRP3 *via* binding with deubiquitinases BRCC3 and JOSD2 (41). In this study, our work showed that MCT4 overexpression in IECs significantly increased ERK1/2-NF-*κ*B activity; blockade of ERK1/2 and NF-*κ*B activity with BAY 11-7085 and U0126 in IECs, respectively, largely attenuated MCT4-induced pyroptosis, suggesting a common mechanism through which MCT4 regulated cell pyroptosis. However, how MCT4 regulated ERK1/2-NF-*κ*B activity would be addressed in our next work. Moreover, the potential function of MCT4 in NLRP3 inflammasome remained to be explored, including ubiquitination, phosphorylation, acetylation, and lactate-mediated acetification. Interestingly, another work from our lab showed that MCT4-mediated metabolic reprogramming, including the metabolic production, is involved in cell pyroptosis, which could be published in our next work. In addition to NLRP3 inflammasomes, whether MCT4 regulated other inflammasome activation such as AIM2 or NLRC4 needs to be address in the next work.

## Conclusion

In summary, we preliminarily revealed a novel function of MCT4 in cell pyroptosis, suggesting that targeting MCT4 expression could improve the treatment of patients with IBD.

## Data Availability Statement

Upon reasonable requirement, the raw data supporting the conclusions of this article will be made available by the authors, without undue reservation.

## Ethics Statement

The studies involving human participants were reviewed and approved by the Medical Ethics Committee for Clinical Ethical Review of Guangzhou Women and Children’s Medical Center. Written informed consent to participate in this study was provided by the participants’ legal guardian/next of kin.

## Author Contributions

WX, JZ, SG, and XZ conceived and designed the experiments. KZ, HC, ZX, JC, YW, HX, XZ, GC, FY, WP, and LH performed the experiments and analyzed the data. KZ, YW, JZ, and WX wrote the manuscript. All authors contributed to the article and approved the submitted version.

## Funding

This work was supported by Natural Science Foundation (KJXW2018071 & 2019RC366), Natural Science Foundation of China (81860101), Natural Science Foundation of Guangdong Province (No. 2017A030313838, No. 2018A0303130175), Guangdong Basic and Applied Basic Research Foundation (No. 2020A1515110109, No. 2021A1515012194), Medical Science and Technology Foundation of Guangdong (No. A2018395, A2021052), Guangzhou Municipal Science and Technology Project (No. 201804010148, No. 201904010485, No. 202102010321), Guangzhou Municipal Health and Family Planning Commission (20201A011039), Funding of Guangzhou Institute of Pediatrics/Guangzhou Women and Children’s Medical Center (No. IP-2016-005, No. IP-2018-009, GWCMC2020-1-005), Funding of Cooperation Projects between Guangzhou Women and Children’s Medical Center and Sun Yat-Sen University (No. 201704020223), and clinical key specialty and construction of cultivating the key subject of Guangzhou Women and Children’s Medical Center (No. 170000105), Scientific Research Initiation Program of Southern Medical University (No. PY2018N050).

## Conflict of Interest

The authors declare that the research was conducted in the absence of any commercial or financial relationships that could be construed as a potential conflict of interest.

## Abbreviations

*α*-CHCA, α-Cyano-4-hydroxycinnamic acid; AMPK, AMP-activated protein kinase; ASC, apoptosis-associated speck-like protein containing a C-terminal caspase recruitment domain (CARD); BRCC3, BRCA1/BRCA2-Containing Complex Subunit 3; CREB, cAMP-response element binding protein (CREB) CD, Crohn’s disease; CDX2, caudal-related homeobox transcription factor 2; DMEM, Dulbecco’s modified eagle medium; ELISA, enzyme-linked immunosorbent assay; ERK1/2, extracellular signal-regulated kinase; FBS, fetal bovine serum; GSDMD, gasdermin D; HMGCS2, hydroxymethylglutaryl CoA synthase 2; IBD, Inflammatory bowel disease; IHC, Immunohistochemistry; IECs, intestinal epithelial cells; IL, Interleukin; JOSD2, Josephin Domain Containing 2; LDH, lactate dehydrogenase; MCT4, Monocarboxylate transporter 4; mTORC, mammalian target of rapamycin complex; NLRP3, NOD-like receptor family, pyrin domain containing 3; NF-*κ*B, nuclear factor kappa-light-chain-enhancer of activated B cells; PCR, Polymerase Chain Reaction; STAT3, Signal transducer and activator of transcription 3; UC, ulcerative colitis; WB, western blot; ZO-1, Zonula occludens-1.
