# Supplementary material for: Monocarboxylate Transporter 4 Triggered Cell Pyroptosis to Aggravate Intestinal Inflammation in Inflammatory Bowel Disease
Source: Front Immunol. 2021 May 19;12:644862. doi: 10.3389/fimmu.2021.644862 (PMC8170300; doi:10.3389/fimmu.2021.644862)
Supplement: Supplementary file 1 [file DataSheet_1.docx]

Supplementary Material

# Supplementary Data

| Case | Sex | Age | type |
| --- | --- | --- | --- |
| Case1 | girl | 12 | healthy control |
| Case2 | girl | 9 | healthy control |
| Case3 | boy | 8 | healthy control |
| Case4 | boy | 6 | healthy control |
| Case5 | boy | 8 | healthy control |
| Case6 | girl | 6 | healthy control |
| Case7 | boy | 8 | healthy control |
| Case8 | girl | 12 | healthy control |
| Case9 | boy | 10 | healthy control |
| Case10 | boy | 7 | healthy control |
| Case11 | boy | 9 | healthy control |
| Case12 | boy | 8 | healthy control |
| Case13 | boy | 9 | healthy control |
| Case14 | boy | 10 | healthy control |
| Case15 | boy | 8 | healthy control |
| Case16 | boy | 7 | IBD(CD) |
| Case17 | girl | 12 | IBD(CD) |
| Case18 | girl | 12 | IBD(UC) |
| Case19 | boy | 9 | IBD(CD) |
| Case20 | girl | 8 | IBD(CD) |
| Case21 | girl | 9 | IBD(UC) |
| Case22 | girl | 8 | IBD(CD) |
| Case23 | boy | 9 | IBD(CD) |
| Case24 | girl | 8 | IBD(UC) |
| Case25 | girl | 9 | IBD(CD) |
| Case26 | boy | 13 | IBD(UC) |
| Case27 | boy | 7 | IBD(CD) |
| Case28 | girl | 11 | IBD(CD) |
| Case29 | girl | 6 | IBD(UC) |
| Case30 | boy | 9 | IBD(CD) |
| Case31 | boy | 7 | IBD(CD) |
| Case32 | girl | 9 | IBD(CD) |
| Case33 | boy | 11 | IBD(CD) |
| Case34 | girl | 8 | IBD(UC) |
| Case35 | girl | 11 | IBD(CD) |
| Case36 | girl | 16 | IBD(CD) |
| Case37 | boy | 10 | IBD(CD) |
| Case38 | boy | 14 | IBD(CD) |
| Case39 | boy | 10 | IBD(CD) |
| Case40 | girl | 13 | IBD(CD) |
| Case41 | girl | 8 | IBD(UC) |
| Case42 | girl | 10 | IBD(CD) |
| Case43 | boy | 8 | IBD(CD) |
| Case44 | girl | 7 | IBD(UC) |
| Case45 | boy | 12 | IBD(CD) |
